# An Overview on Image Registration Techniques for Cardiac Diagnosis and Treatment

**DOI:** 10.1155/2018/1437125

**Published:** 2018-08-08

**Authors:** Azira Khalil, Siew-Cheok Ng, Yih Miin Liew, Khin Wee Lai

**Affiliations:** ^1^Department of Biomedical Engineering, Faculty of Engineering, University of Malaya, 50603 Kuala Lumpur, Malaysia; ^2^Faculty of Science and Technology, Islamic Science University of Malaysia, 71800 Nilai, Negeri Sembilan, Malaysia

## Abstract

Image registration has been used for a wide variety of tasks within cardiovascular imaging. This study aims to provide an overview of the existing image registration methods to assist researchers and impart valuable resource for studying the existing methods or developing new methods and evaluation strategies for cardiac image registration. For the cardiac diagnosis and treatment strategy, image registration and fusion can provide complementary information to the physician by using the integrated image from these two modalities. This review also contains a description of various imaging techniques to provide an appreciation of the problems associated with implementing image registration, particularly for cardiac pathology intervention and treatments.

## 1. Introduction

Cardiovascular disease (CVD) remains the leading cause of death around the world. It accounts for an estimated 17.7 million deaths in 2015, representing 31% of all global deaths [1].  Table 1 shows a percentage breakdown of the deaths due to CVDs around the globe. As shown in the table, 8.7 million were attributed to ischemic heart disease and 6.2 million to stroke. Rheumatic heart disease, such as heart valve disease, accounts for 0.3 million deaths. Hypertensive heart diseases, such as heart failure, thickening of the heart muscle, and coronary artery disease, account for 0.9 million deaths. Moreover, 0.4 million deaths were attributed to inflammatory heart diseases, such as cardiomyopathy, myocarditis, and endocarditis. Approximately 1.0 million deaths were due to other circulatory diseases. These epidemiologic data show that CVD is today the largest single contributor to global mortality, and the World Health Organization estimates that CVD will continue to dominate mortality trends in the future. Thus, additional efforts should be aimed at diagnosing and treating the CVD to improve patient's health and decrease this alarming mortality rate.

Medical imaging plays a vital role in the diagnosis of cardiac diseases. There are several imaging modalities that can diagnose cardiac diseases, for example, X-ray, echocardiography, single-photon emission computed tomography (SPECT), positron emission tomography (PET), CT, and magnetic resonance imaging (MRI). Imaging the heart with a single modality will provide the physician with insufficient information regarding the heart's condition. The heart is indeed a nonrigid but dynamic structure. Heart deformation inevitably occurs during the cardiac pumping cycle. As each imaging modality provides unique information and overcomes only certain challenges in cardiac imaging, the physician usually prescribes more than one imaging procedure to gather as much information of the heart's condition before making a treatment decision. Imaging modality also plays important roles during interventional cardiac treatment. Some imaging modalities, such as echocardiography and fluoroscopy/angiography, are often used during interventional procedures to provide visual aid to the physician. In certain clinical cases, multiple images are acquired at different time points or from different viewpoints. Images can also be taken with differing imaging modalities. Thus, image fusion is often useful for the integration of various sources of information. It involves a principal step of image registration.

In the biomedical research field, image registration is an ongoing research. In 1992, Brown [2] performed a comprehensive survey on different registration methods. This was followed by thorough reviews describing the state-of-the-art registration algorithms and applications by Maintz and Viergever [3], Audette et al. [4], Hill et al. [5], and Zitová and Flusser [6]. Lester and Arridge [7] and Hill et al. [5] provided reviews focusing on image registration in the medical field, whereas Makela et al. [8] and Zitová and Flusser [6] provided reviews on cardiac image registration. Multimodality image registration of cardiac structures is a complicated process relative to the fusion of other body parts, such as the brain or kidney, due to the heart and thorax motions as well as the deformable nature of the heart. This study aims to provide an overview on image registration research performed using various imaging modalities, purpose of image registration in cardiovascular imaging, and implementation strategies of image registration.

## 2. Medical Imaging Modalities for Cardiac Imaging

### 2.1. Chest X-Ray

Chest X-ray (CXR) examination is almost often prescribed as the first imaging procedure for a patient with suspected cases of heart or lung disease. It is a noninvasive imaging procedure where a small amount of X-ray radiation is used to expose the chest in creating a static projection image on the detector [9]. CXR provides structural information, including the location, size, and shape of the heart as well as the lungs and bones of the chest. However, it cannot determine the internal heart structure due to the loss of depth information. Therefore, heart disease cannot be accurately diagnosed based on CXR alone. Current advances in dual-energy (DE) CXR have many advantages over conventional chest radiography, facilitating cardiac image interpretation. With additional techniques of image registration, coronary artery calcium can be identified using DE CXR [10, 11]. However, the use of radiation in the imaging procedure also confounds the use of this modality for real-time imaging; therefore, no functional information of the heart can be obtained.

### 2.2. Angiography

Angiography is another imaging modality that also utilizes X-ray radiation to image the heart. This procedure requires injection of a radiocontrast agent into the blood vessel, and a low-dose X-ray beam is used to create real-time images of the heart based on fluoroscopy [12]. In ischemic heart disease such as coronary artery disease, angiography can be used to identify the infarct region [13, 14]. In addition, this imaging procedure is used for left ventricular (LV) [15] lead implantation during cardiac resynchronization therapy for patients suffering from heart failure [16–18]. Angiography is also useful for guiding the physician during cardiac electrophysiology procedures for patients with cardiac arrhythmias [19–21]. Furthermore, angiography can be applied to congenital heart disease patients during cardiac catheterization procedures [22, 23]. However, this procedure is contraindicated for patients who are allergic to contrast agents and is a high risk for hypertensive patients. Furthermore, due to the radiation exposure over an extended period of time during the procedure, it is also not recommended for pregnant women and children due to the risk of radiation.

### 2.3. Myocardial Perfusion Imaging (MPI)

MPI is another noninvasive type of heart examination that uses nuclear medicine imaging techniques such as SPECT and PET [24]. This imaging procedure uses a small quantity of radioactive materials (radiotracer) that are typically injected into the blood vessel, inhaled, or swallowed. The radiotracer travels to the target area and gives off gamma rays, which are detected by a gamma camera and computer to process the images of the examined structure. MPI provides information on blood flow and heart function for the detection of coronary artery disease as well as the extent of coronary stenosis [25]. It also assesses the damage to the heart following a heart attack [25]. SPECT can also be used during LV tachycardia ablation procedures [26] for patients with cardiac arrhythmias. Other than detecting heart diseases, SPECT and PET are also used to improve attenuation correction [27–29] and respiratory motion during MPI [15, 25, 30–39]. However, MPI is limited by poor system resolution, and localization of the affected anatomical site has been reported to be difficult [40].

An important advance in the design of SPECT and PET scanner is the introduction of hybrid SPECT-CT/MR [41, 42] and PET-CT/MR machines [41, 43]. These hybrid machines allow examinations to be performed while the patient remains in the same examination table. Thus, this scanner reduces the imaging time, which interprets into increased patient comfort and cooperation. The hybrid scanner uses CT or MR images as transmission images for attenuation correction of the SPECT or PET imaging. The scanner then performs registration of the transmission images with the SPECT or PET images immediately after the CT or MRI. After image reconstruction, both imaging data are displayed either side by side or overlaid. The physician can gain both functional information by SPECT or PET imaging and accurate anatomical information of abnormal metabolic activity by CT or MR examination. Although these hybrid machines are designed to inherently solve the image fusion between imaging, additional image registration procedures are still implemented if severe movement of patients occurs between the SPECT or PET and CT or MR imaging [43].

### 2.4. CT

Cardiac CT produces cross-sectional images of the heart structure. It represents the X-ray attenuation properties of the tissue being imaged [44]. Thin X-ray radiations are used to scan the FOV and yield line attenuation measures at all possible angles of the body being imaged. The line attenuation measures are used to reconstruct the 3D attenuation map of the body with each point on the map carrying the value of X-ray attenuation coefficient. Spiral or sequential technique can be used to reconstruct the 3D image of the heart. Additionally, contrast agents can also be used to enhance the visualization of blood vessels and identify the presence of tumors because of the difference in contrast uptake between tumors and surrounding normal tissue.

Cardiac CT is commonly applied as a preoperative imaging tool, while multi-slice CT angiography (CTA) is a promising technology for imaging patients with suspected coronary artery disease [45]. In addition, CT and CTA are widely used for calcium scoring for patients with the risk of coronary heart disease [46]. Recent advances in deformable registration techniques have enabled CT scan to identify regional infarction in heart disease patients [47] and aneurysm morphodynamics [48]. Furthermore, volumetric CT scans also provide essential information critical in treatment planning, including the morphology of the valves and stenosis severity [49–51]. However, cardiac CT cannot be utilized as a real-time intraprocedural guidance due to the use of high-dose ionizing radiation. It also cannot provide information on hemodynamics such as transvalvular pressure gradients and cannot detect valve regurgitation [52].

### 2.5. MRI

Similar to cardiac CT, cardiac MRI can also provide 3D cross-sectional images of the heart. MRI examination utilizes strong magnetic field to produce maps of atomic nuclei (hydrogen atoms in water or fat molecules in the body) [53]. The spin of atomic nuclei can be considered as a magnetic vector, causing the proton to behave like a magnet. The image acquisition involves an initial sequence of exciting pulses and recording of the emitted signal. The amplitude of the signal is then used to generate maps showing the heart structures. MRI produces images with high resolution as well as high tissue contrast useful for the assessment of heart chambers [54–57], heart valves [58, 59], size and blood flow through the major vessels [60], and surrounding structures such as the pericardium [61]. MRI is also utilized in diagnosing a variety of cardiovascular disorders such as tumors [62, 63] and inflammatory conditions.

Research has also been performed to evaluate the effects of coronary artery diseases, such as limited blood flow to the heart muscle and scarring within the heart muscle followed by heart attack [64, 65], using MRI. In addition, MRI can be utilized for the preoperative treatment planning procedure [66] and monitoring the progression of certain disorders over time [67, 68]. Furthermore, MRI can evaluate the anatomy of the heart and blood vessels [69, 70] as well as the effect of surgical changes [71, 72], especially in patients with congenital heart diseases [59, 61]. In addition, MRI provides information about cardiac necrosis and fibrosis through late gadolinium-enhanced imaging, iron overload measurement, and myocardial tissue characterization by relaxometry [73]. However, compared with cardiac CT, these examinations are much more expensive and contraindicated for patients with metallic implants such as graft stents, cardiac pacemaker devices, or hemodynamic support devices. Although MRI is the gold standard for cardiac assessment, its resolution is still insufficient to resolve valve leaflets. Therefore, MRI is not feasible for surgical planning of valvular diseases.

### 2.6. Echocardiography

Echocardiography is the ultrasound (US) imaging of the heart. It is a vital imaging examination for diagnosing heart disease. The basic working principle of echocardiography involves the generation of high-frequency sound waves by a US probe, which is directed toward the tissue. A portion of the sound waves that penetrates the tissue are reflected back toward the transducer when the waves encounter boundaries of tissues with different reflective indices. The reflected signals are detected and processed by the echocardiography system to reconstruct images of the heart structures. Currently, two-dimensional (2D) echocardiography is the main preoperative imaging modality for cardiac pathologies.

In valvular disease, this modality allows clinicians to estimate the degree of valve regurgitation or stenosis, valve annular size, involvement of the leaflets, chordal and papillary muscle structural integrity, and overall LV size and LV systolic function [74–77]. Echocardiography also plays a significant role in hypertensive patients as it provides information on LV mass [15], LV systolic function, impaired LV diastolic function, and left atrial (LA) size and function [78, 79]. In addition, coronary artery disease can also be detected using this intravascular US (IVUS) technology [80, 81]. Echocardiography provides screening for early cardiac disease detection and intervention for rheumatic heart disease and ischemic heart disease [82–84]. It can also be used for intraoperative image guidance to facilitate physicians in surgery due to its real-time capability without the use of ionizing radiation [55, 85–95]. It is also a low-cost option relative to other imaging modalities [96]. However, the quality of echocardiography images is inferior compared with other imaging modalities such as cardiac CT and MRI due to the presence of speckle noise and limited field of view (FOV) [97].

Another type of echocardiography is transesophageal echocardiography (TEE). TEE provides detailed images of cardiac structures near the upper chambers, such as heart muscle and chambers, valves, pericardium, and blood vessels connected to the heart. It is also utilized during cardiac treatment procedures to assist device positioning and deployment. However, compared with 2D echocardiography, TEE is an invasive procedure because images are acquired by passing a thin tube attached with an echo probe through the patient's mouth, down the throat into the esophagus [98]. Furthermore, due to the long duration of cardiac treatment, continuous TEE monitoring usually requires general anesthesia with endotracheal intubation. By contrast, intracardiac echocardiography (ICE) can be prescribed to aid such procedures. Similar to the technique of IVUS, the ICE is a catheter-based imaging that provides images within the heart. Compared with the limited view provided by TEE, the echo probe attached to the catheter in ICE can be maneuvered within the heart, thus providing more detailed images within the heart as well as aortic root images. Unlike TEE, ICE requires only local anesthesia, which reduces the risk and discomfort of endotracheal intubation, esophageal intubation, and general anesthesia associated with TEE [99].

Three-dimensional (3D) echocardiography can provide real-time 3D visualizations of the heart structure and overcome some of the limitations of conventional 2D echocardiography [74, 79, 94]. Unfortunately, such technology is not widely available and expensive compared with 2D echocardiography.

## 3. Purpose of Image Registration in Cardiac Imaging

The purpose of image registration is to align images with respect to each other. The result of image processing can help in further medical image analysis for various purposes, including correlating clinical features from different cardiac images, respiratory motion correction, facilitating the cardiac segmentation procedure, complementary information for image fusion, and image guidance for therapeutic intervention.

### 3.1. Facilitation of Image Segmentation

Cardiac analysis includes the calculation of structural and functional cardiac indices. Such indices include chamber volumes, stroke volume, ejection fraction, cardiac output, myocardial mass, and myocardial wall thickness and thickening. These indices can be calculated by segmenting the heart chambers [100]. However, delineating the heart manually on multiple slices and frames requires a considerable amount of time. Furthermore, this is subject to well-established intra- and intersubject variability. Thus, current advances in image registration techniques have adopted automated cardiac segmentation techniques that can rapidly, objectively, and accurately extract the chamber boundaries from medical images in clinical practice. Among the four chambers, the LV has received the most attention in image registration for cardiac segmentation [21, 57, 64, 101–110]. This is because it plays a key role on the process of blood circulation, and thus, its function/dysfunction is associated with most cardiac diseases. Furthermore, the LV has a relatively simple geometry with thick myocardial walls, making its automated segmentation more feasible. Compared with LV, studies on image registration for the segmentation of the right ventricle (RV) [107, 111] and LA [65, 112, 113] are few due to the more complex geometry of these chambers and their much thinner walls. However, these chambers are associated with many critical diseases, such as modeling in patients with pulmonary hypertension [111, 114] or LA enlargement [65, 112, 113, 115, 116]. Further research is thus required to develop techniques capable of coping with the difficulties of segmenting complex RV and right atrial walls and to segment the whole heart to enable an assessment that takes into account the combined motion of all chambers.

### 3.2. Respiratory Motion Correction

Dynamic motion of the heart, spatial errors due to systole and diastole, and phasic changes during respiration can cause changes in size and position of the heart during cardiac imaging, thus significantly impairing the image quality and diagnostic accuracy of cardiac image analysis. Gating to both the cardiac cycle and respiration can potentially help to reduce these errors. However, another source of error due to varying patient positions between imaging modalities can still produce motion artifacts during the cardiac imaging and interventional procedure. Cardiac motion can be estimated using the deformable registration scheme performed on initial images of different cardiac phases. This motion information can be used for a motion-compensated reconstruction, allowing the use of all acquired data for image reconstruction [117]. The image registration plays an important role in motion correction during coronary angiography [117–120], SPECT [15, 27, 35–38, 121], PET [28, 122–125], CT [31, 126–129], MRI [56, 130–135], and echocardiography [136, 137]. The respiratory motion also allows the use of predictive models that compute information about the respiratory cycle and use them for image registration techniques [138].

### 3.3. Direct Surgical Guidance

Minimally invasive transcatheter cardiac interventions are being adopted rapidly to treat a range of CVDs such as valve repairs and implantation [76, 87, 88, 90, 91, 93, 139, 140], atrial fibrillation therapy, and bypass surgery [141]. 2D echocardiography and fluoroscopy/angiography have been used as intraoperative imaging modalities to provide surgical guidance to clinicians owing to their real-time in vivo functionality. Despite the aforementioned advantage, the image quality of echocardiography is confounded by speckle noise and limited FOV, therefore appearing inferior compared with other preoperative modalities such as CT or MRI [97]. Moreover, 2D echocardiography and fluoroscopy/angiography provide only planar images. Issues associated with maintaining both the surgical tool and the anatomical targets in the same view during surgery have also been reported [142]. By contrast, the preoperative imaging modalities such as CT and MRI play an important role in surgical planning and simulation of cardiac interventions. Overlaying a 3D cardiac model extracted from preoperative images onto real-time echocardiography or fluoroscopic images provides valuable visual guidance during the intervention. The principal step in this integration involves image registration techniques. As both images were acquired at different times and have distinct image properties, image registration can be applied to temporally and spatially align both images, thus providing better information during surgical guidance. Such integration has been applied using 2D echocardiography-CT [87, 90, 91, 93, 143, 144], 3D echocardiography-CT [91], and 2D echocardiography-MRI [145].

### 3.4. Image Fusion

Image registration is a principal step in image fusion. Image fusion can provide clinicians with complementary information from the integrated information from each modality. For example, echocardiography can provide functional information such as functional information of cardiac valves, valve regurgitation, stenosis, and systolic function and measure systolic ejection performance and the extent of wall hypertrophy. However, the image quality of echocardiography is inferior to other 3D imaging modalities due to the presence of speckle noise and limited FOV. The 3D imaging modalities such as CT and MRI can provide quantitative assessments on valve morphology, valvular dysfunction severity, and structural information of chambers and vessels. Thus, establishing direct spatial registration between these modalities can provide physicians with functional and structural information crucial to diagnosing and treatment planning of cardiac diseases [55, 88–95, 146, 147]. The registration of SPECT-CT [148] and PET-CT [149–151] also facilitates information fusion of both modalities. Molecular and functional PET and SPECT imaging are used to assess myocardial perfusion, perfusion reserve, metabolism, and innervation. With registration to CT, the calcification of coronary arteries can be detected and quantified.

## 4. Implementation Strategy

**Figure 1:**
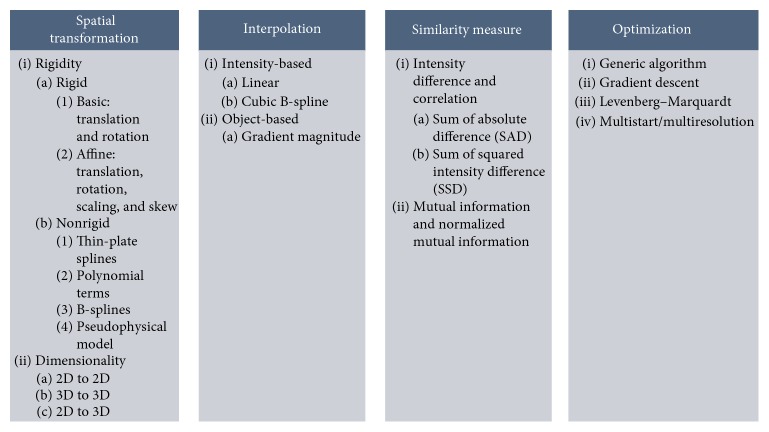
Different methods in the implementation strategy for cardiac image registration.

Image registration is a process that finds a transformation that maps features in one coordinate space to their location in another coordinate space [152]. It allows one to transfer information between both coordinate spaces. The basic steps in the image registration algorithm often involve feature extraction, geometrical transformation definition, similarity measure, and optimization. First, the feature space extracts the selected feature to be used for mapping. Second, the search space determines the degree of transformation that brings alignment between the fixed and moving images. Let us assume that there are two images to be spatially registered: the moving image, *R*(*x*), which is defined over a domain *x* ∈ *V*_R_, and the fixed image, *S*(*x*), which is defined over a domain *x* ∈ *V*_S_. Transformation, *T*(*x*), is used to transform points in *R*(*x*) to their corresponding positions in *S*(*x*). The commonly used transformations include rigid, affine, projective, and curved transformations. A similarity measure is used to estimate the similarity merit of the fixed image and transform the moving image. The search for optimal transformation parameters to register the images spatially can be accomplished by optimizing (maximizing or minimizing) a similarity measure derived from features in the image, for example, the intensities of voxels in the images. The search process continues according to the optimization strategy until convergence, that is, transformation parameters that minimize or maximize the similarity measure are found [152, 153].  Figure 1 shows an overview of implementation strategy approaches.

### 4.1. Spatial Transformation

#### 4.1.1. Rigid and Nonrigid Transformation

In choosing the types of spatial transformation algorithms, assumptions on heart rigidity are generally made. In most cases, the heart is assumed to be a rigid body structure, where no changes or deformations occur from the time of imaging to the time of registration [79, 104, 154–164]. Rigid spatial transformation usually assumes the heart to be rigid with periodic heart motion throughout the imaging process. In rigid transformation methods, the entire 2D or 3D images are transformed. The basic rigid transformation methods include six degrees of freedom (or unknowns) in the transformation: three translations and three rotations. Another rigid transformation method that includes scaling and skew parameters is the affine transformation method. The affine rigid transformation can include up to nine degrees of freedom (three translations, three rotations, and three scaling parameters) or 12 degrees of freedom (three translations, three rotations, three scaling parameters, and three skews).

The rigid spatial transformation [79, 104, 154–164] in the registration framework is commonly used in clinical practice and is considered to be acceptable for reaching the correct diagnosis [151]. Although this hypothesis is valid in some surgical scenarios, the heart is indeed a nonrigid but dynamic structure. Deformation of the heart inevitably occurs during the pumping cycle. Other factors such as respiration and probe and surgical instrument pressure on the skin can also contribute to the deformation of the heart. These factors can jeopardize the accuracy of image registration during real-time imaging.

Meanwhile, nonrigid spatial transformation utilizes nonaffine registration algorithms [79, 104, 154–164]. In some cases, the nonaffine transformation is applied after initial estimation given by rigid body or affine transformation [27, 123, 165–169]. Thin-plate splines are often used to determine the transformation [170–179]. Using intensity-based algorithms, the nonrigid component of the transformation can be determined using a linear combination of polynomial terms [120, 180], basis functions, or B-spline [27, 49, 80, 181–184] surfaces defined by a regular grid of control points. As an alternative, pseudophysical models, such as elastic deformation or fluid flow [78, 169, 185–187], can be used, wherein the deformation between the images is modeled as a physical process. Some registration techniques also include a mixture of rigid and nonrigid transformations in the same algorithm such as nonaffine registration [156].

#### 4.1.2. Dimensionality

Selection of spatial transformation algorithms also depends on the image dimensionality, whether involving the registration from 2D to 2D [10, 11, 136, 171, 183], 3D to 3D [22, 49, 74, 78, 139, 162, 179, 188, 189], or 2D to 3D [79, 92, 93, 143, 190, 191]. For 2D to 2D registration, where the acquisition tightly controls the geometry of the images, the images can simply be registered via a rotation and two orthogonal translations. In addition, the scaling factor may also be considered from the real object to each of the images. Meanwhile, 3D to 3D registration is used to align tomography datasets or a single tomography image to any spatially defined information. This registration is based on the assumption that the internal anatomy of the patient is not distorted or changed in spatial relationships between organs. The 3D to 3D registration requires calibration on each scanning device to determine the scale of the scanned images. However, the registration of 2D to 3D images is more complex than that of 2D to 2D or 3D to 3D images. It involves establishing the concurrence between 3D volumes such as CT or MRI and planar images such as X-ray or optical images. Furthermore, they are needed when the position of one or more slices from tracked B-mode US, interventional CT, or interventional MR images is to be constructed regarding 3D volume. The 2D to 3D registration often has computational complexity, and the speed of registration is considered a problem.

### 4.2. Interpolation

The choice of interpolation method also plays a significant role during the image registration process. The interpolation method will sample input image in the same size, spatial location, and orientation as the image to be registered. Two types of interpolation methods exist: intensity-based interpolation and object-based interpolation. In intensity-based interpolation, the final result from the interpolation is directly computed from the intensity values of input images. Examples of intensity-based interpolation include linear [92, 93, 162, 192] and cubic B-spline [27, 49, 80, 181–184] interpolation methods. Given its computational simplicity, intensity-based interpolation has been widely used in cardiac image registration research. The resultant image is basically a weighted average of input image. However, the main limitation of these methods is the blurring effects on cardiac structure boundaries.

Meanwhile, in object-based interpolation, the extracted information from objects contained in input images is used to guide the interpolation into more accurate results. One of the object-based interpolation techniques is the gradient magnitude-based approach, which involves finding corresponding points between consecutive slices and then applying the linear interpolation to find in-between slices. In this technique, a small difference between consecutive slices is assumed; thus, the search for correspondence points to small neighborhoods is restricted. However, the limitation is that this assumption is not applicable in many cases. To reduce the blurriness of edges, more recent approaches have been studied, including the polynomial curve fitting [120, 180] method.

### 4.3. Similarity Measure

Similarity measures can be based on intensity difference and correlation methods or based on the mutual information method. Registration based on image intensity difference and correlation methods [129, 190, 193–197] is best for intramodality image registration because the method measures the similarity between images that differ primarily because of different image acquisition conditions (such as noise). For example, a study used the sum of absolute difference (SAD) and sum of squared intensity difference (SSD) similarity measures [79]. In the SAD method, registered images are subtracted pixel by pixel, and the mean value of the sum of the absolute intensity difference of all pixels in the subtracted image is computed. The SSD is similar to the SAD measure, but it is calculated instead of the absolute difference.

Registration based on mutual information or normalized mutual information [92, 93, 109, 131, 136, 139, 189, 198–204] uses a similarity metric in the registration process. This similarity measure is arguably an excellent similarity metric and has been shown to be robust and well suited for multimodal image registration. The method compares the information or entropy between images to arrive at its measure. The mutual information assumes that when images are perfectly registered, their shared entropy is minimized.

### 4.4. Optimization

To register the images, an optimal value of the similarity measure over a parameter space with dimensionality defined by the number of degrees of freedom of the transformation is calculated. The optimization algorithm then makes another estimate of the transformation, evaluates the similarity measure again, and continues until the algorithm converges, at which point no transformation can be found that results in a better value of the similarity measure within a preset tolerance. Each registration algorithm that makes use of a voxel similarity measure tends to use a different optimization algorithm.

One of the optimization techniques used in cardiac image registration is genetic algorithms, which are known to be very robust for searching and optimization. It is based on the principles of natural biological evolution, which operates on a population of potential solutions applying the principle of survival of the fittest to produce better approximations to a solution. One of the examples of genetic optimization algorithms is the generalized pattern search (GPS) [92, 93]. The GPS algorithm does not involve the gradient of the objective function to be optimized; thus, it is specifically suited for function that is not differentiable, such as mutual information in its usual form. Other optimization methods include the gradient descent optimization method [80, 197] and Levenberg–Marquardt optimization method [160, 205] in minimizing the variance in intensities of corresponding pixels.

In addition, a multistart or multiresolution optimization method [80, 206–208] can be utilized to the registration workflow. These methods are one way to explore the transformation parameter space. Combined with other local optimization methods, they restart the search for the global optimum from a new solution once a region (or a path) has been explored by local optimization. The new start position is uniformly sampled from the transformation parameter space.

## 5. Conclusion

In this study, various imaging modalities and their purposes and implementation strategies in image registration for cardiac diagnosis and treatment are presented. Many imaging modalities are used for diagnosing cardiac diseases. Each imaging modality provides unique information and overcomes certain challenges in cardiac imaging. Multimodality imaging can provide physicians with more information of the heart's condition before making a treatment decision. The integration of image fusion from various types of imaging modalities involves a principal step of image registration. As the intent of image registration is to align images with respect to each other, image registration can assist physicians for further medical image analysis for various purposes, including correlating clinical features from different cardiac images, respiratory motion correction, facilitating the cardiac segmentation procedure, complementary information for image fusion, and image guidance for therapeutic intervention.

In the implementation strategy of cardiac image registration, the basic steps include feature extraction, geometrical transformation definition, and similarity measure and optimization. Although previous studies have achieved image registration goals, no single standard algorithm can be applied in aligning two modalities. The multimodality registration algorithms need to take into account factors such as heart rigidity, dimensionality of the image dataset, interpolation methods, types of similarity measures, and choices of optimization approaches. However, challenges remain in overcoming the deformation of the heart and the respiratory motion, which can affect the accuracy of image registration, especially during real-time imaging for guiding the treatment procedure. Although significant work has been made in addressing such an issue, there is much more room for the development of fully automatic cardiac image registration by taking into account heart deformation and respiratory motion in the future.

## Figures and Tables

**Table 1:** Percentage breakdown of deaths due to CVDs in 2015 [1].

Types of CVD	Percentage of death (%)
Rheumatic heart disease	1.73
Hypertensive heart disease	5.33
Ischemic heart disease	49.50
Stroke	35.30
Ischemic stroke	16.46
Hemorrhagic stroke	18.82
Cardiomyopathy, myocarditis, and endocarditis	2.34
Other circulatory diseases	5.83

## Data Availability

The data supporting this review are obtained from previously reported studies and datasets, which have been cited within the manuscript as references.
